# *Streptococcus agalactiae* Inhibits *Candida albicans* Hyphal Development and Diminishes Host Vaginal Mucosal TH17 Response

**DOI:** 10.3389/fmicb.2018.00198

**Published:** 2018-02-23

**Authors:** Xiao-Yu Yu, Fei Fu, Wen-Na Kong, Qian-Kun Xuan, Dong-Hua Wen, Xiao-Qing Chen, Yong-Ming He, Li-Hua He, Jian Guo, Ai-Ping Zhou, Yang-Hong Xi, Li-Jun Ni, Yu-Feng Yao, Wen-Juan Wu

**Affiliations:** ^1^Department of Laboratory Medicine, Shanghai East Hospital, Tongji University School of Medicine, Shanghai, China; ^2^Department of Obstetrics and Gynecology, Shanghai East Hospital, Tongji University School of Medicine, Shanghai, China; ^3^Institut Pasteur of Shanghai, Chinese Academy of Sciences, Shanghai, China

**Keywords:** *C. albicans*, *S. agalactiae*, hyphal development, vaginal mucosa, TH17 response

## Abstract

*Streptococcus agalactiae* and *Candida albicans* often co-colonize the female genital tract, and under certain conditions induce mucosal inflammation. The role of the interaction between the two organisms in candidal vaginitis is not known. In this study, we found that co-infection with *S. agalactiae* significantly attenuated the hyphal development of *C. albicans*, and that *EFG1*-Hwp1 signal pathway of *C. albicans* was involved in this process. In a mouse model of vulvovaginal candidiasis (VVC), the fungal burden and the levels of pro-inflammatory cytokines, IL-1β, IL-6 and TNF-α showed a increase on co-infection with *S. agalactiae*, while the level of TH17 T cells and IL-17 in the cervicovaginal lavage fluid were significantly decreased. Our results indicate that *S. agalactiae* inhibits *C. albicans* hyphal development by downregulating the expression of *EFG1*-Hwp1. The interaction between *S. agalactiae* and *C. albicans* may attenuate host vaginal mucosal TH17 immunity and contribute to mucosal colonization by *C. albicans*.

## Introduction

*Candida albicans* is a common opportunistic pathogen that colonizes human mucosal surfaces. It is a major cause of opportunistic infections ranging from superficial to fatal systemic infections (Calderone, 2002). In immunocompromised patients, colonies of *C. albicans* can lead to recurrent mucosal and life-threatening disseminated infections that are associated with a high mortality (Tancrede and Andremont, 1985; Perlroth et al., 2007). Generally, *C. albicans* co-exists with numerous bacterial species in different host niches, such as skin and vaginal mucosa. The combination of physical association and molecular interaction between bacteria and fungi can result in different outcomes. Recent evidence indicates that bacteria play an important role in the pathogenesis of infection with *C. albicans* (Sztajer et al., 2014).

*Staphylococcus aureus, Lactobacillus* sp., *Enterococcus* and other bacteria have been shown to alter the biological activity *of C. albicans* (Morales and Hogan, 2010; Cruz et al., 2013; Nash et al., 2014). *Pseudomonas aeruginosa* and *C. albicans* are antagonistic *in vitro*; the bacteria damage fungal hyphae and the fungal organism reverts to a resistant yeast form in response to prokaryotic quorum-sensing factors (Hogan et al., 2004; Brand et al., 2008; Shirtliff et al., 2009; Purschke et al., 2012). The interaction of *C. albicans* and *P. aeruginosa* not only with their host, but also with each other is evidently multifaceted (Fourie et al., 2016). In oral mucosal infection, *Streptococcus mutans* exhibited high affinity for *C. albicans* hyphae (Metwalli et al., 2013). *Streptococcus oralis* and *C. albicans* have been shown to synergistically activate μ-calpain to degrade E-cadherin from oral epithelial junctions (Xu et al., 2016). In the vagina, lactic acid bacteria are known to suppress filamentation of *C. albicans* by producing lactic acid and other metabolites (Hutt et al., 2016). Lactic acid bacteria differentially regulate *C. albicans* filamentation in white and opaque cell types under different environmental conditions (Liang et al., 2016). Up to 50 different bacterial species have been isolated as part of the normal or abnormal vaginal microbiota (Gajer et al., 2012). Co-infection of female genital tract with *S. agalactiae* and *C. albicans* in patients with polymicrobial aerobic vaginitis was shown to be associated with an increased risk of preterm delivery (Donders et al., 2009; Mendling, 2016). When *C. albicans* infects host mucosa, hyphae predominate at the primary site of infiltration in the epithelial tissue, whereas yeast cells are generally found either on the epithelial cell surface or emerge from the infiltrating hyphae. Tissue macrophages, neutrophil and dendritic cells then sample the contents of the microbial flora on the mucosa and induce host immune response (Fidel et al., 1999; Nomanbhoy et al., 2002; De Bernardis et al., 2006; Lionakis et al., 2011). Though limited information is available about the immune mechanisms operating in the vaginal mucosa, it is quite clear that the vaginal mucosa has immunocompetent cellular components that are capable of eliciting innate immune responses and that TH17 T cells are possibly independent of the systemic immune response (Harrington et al., 2005; Conti et al., 2009; Pietrella et al., 2011).

Aerobic vaginitis is associated with aerobic micro-organisms, mainly group B streptococci and *Escherichia coli*. Its characteristics are different from those of bacterial vaginosis and elicit a host immune response that leads to high production of IL-6, IL-1β, and leukemia inhibitory factor in the vaginal fluid (Donders et al., 2002). In pregnant patients with aerobic vaginitis, *C. albicans* was always isolated from vaginal secretions along with *S. agalactiae*, *Staphylococcus aureus*, and *E. coli* (Donders et al., 2011). In our laboratory, *S. agalactiae* and *C. albicans* were commonly isolated from patients with recurrent vulvovaginal candidiasis (RVVC). *S. agalactiae* and *C. albicans* were co-isolated from clinical specimens of 5% of patients with RVVC. These observations prompted us to elucidate the interactions between *C. albican*s and *S. agalactiae* in the context of vaginal mucosal infection. *C. albicans* is a dimorphic yeast and its ability to switch its morphology between yeast and hyphal forms is crucial for colonizing tissues and in infection (Saville et al., 2003; Han et al., 2011). In this work, we discovered that *S. agalactiae* inhibited *C. albicans* hyphal morphogenesis and the *S. agalactiae* inhibits *C. albicans* hyphal development by inhibiting *EFG1*/Hwp1 expression. Furthermore, experiments on mice showed that this co-infection decreased the TH17 T cells immune response without innate immune response downregulation. These findings demonstrate that the biological interaction between *S. agalactiae* and *C. albicans* may weaken the immune response of the TH17 T cells in the vaginal mucosa.

## Materials and Methods

### Strains and Growth Conditions

*Streptococcus agalactiae* strain was isolated from patients with RVVC and named Saga-Eh1. *C. albicans* strain SC5314, *C. albicans* SC5314-GFP and *S. agalactiae* strain Saga-Eh1 were stored in our laboratory. *C. albicans* strain was cultured on Sabouraud dextrose chloramphenicol agar plate and then maintained in yeast extract peptone dextrose (YPD) medium at 30°C. Saga-Eh1 was cultured on blood plate or Tryptone soya broth (TSB) medium at 37°C (**Table 1**).

**Table 1:** Bacterial/fungi strains used in this study.

Strains	Relevant property	Source
SC5314	Prototroph	Fonzi and Irwin (1993)
SC5314-GFP	*C.albicans* SC5314 carrying the GFP reporter gene	This study
CA-RVVC	*C. albicans* strains isolated from patients of RVVC infection	This study
CA-RVVCS	*C. albicans* strains isolated from patients of RVVC infection without bacterial infection	This study
CA-RVVCC	*C.albicans* strains isolated from patients of RVVC with *S. agalactiae* co-infection	This study
Saga-Eh1	*S. agalactiae* clinical isolate	This study
*E. coli* DH5a	*E. coli* cloning host for routine cloning	Invitrogen


### *S. agalactiae* Identification

Clinically isolated *S. agalactiae* strains were identified using VITEK2 COMPACT (BIOMERIEUX), a single colony selected for future identification. PCR amplicons of *S. agalactiae* 16S ribosomal DNA (16SrDNA) (Forward primer:AGTTTGATCCTGGCTCAG; Revese primer:GGTTACCTTGTTACGACTT) identification were obtained from chromosomal DNA. The sequences of the PCR products were compared with the existing sequences available at https://blast.ncbi.nlm.nih.gov. To further confirmation isolated clinical *S. agalactiae* strains CAMP detection were positive (**Table 1**).

### *C. albicans* Identification

Clinically isolated *C. albicans* strains were identified using VITEK2 COMPACT (BIOMERIEUX), and a single colony selected for future identification. PCR amplicons of *C. albicans* the internal transcribed spacer (ITS) region (ITS1-ITS4) (ITS1primer:TCCGTAGGTGAACCTGCGG; ITS4 primer:TCCTCCGCTTATTGATATG) were obtained from chromosomal DNA. The sequences of the PCR products were compared with the existing sequences available at https://blast.ncbi.nlm.nih.gov (**Table 1**).

### Hyphal Induction Assays

Hyphal induction was manipulated using a standard method. Strains were grown overnight in liquid YPD at 30°C, and diluted 1:250 in Spider media, YPD+10% fetal bovine serum (FBS), SD+10% FBS and Roswell Park Memorial Institute (RPMI) 1640. Cells were incubated at 37°C and harvested, and hyphal morphology were examined under a microscope at the indicated time points.

### *Ex Vivo* Transwell Co-culture Assay

*Candida albicans* cells were added to the upper part of the Transwell chamber (3 μm pore, Costar), and *S. agalactiae* cells were added to the lower chamber, which allowed us to observe hyphal development of *C. albicans* at 37°C toward active molecule-containing media or cell-conditioned media at different time points.

### RNA Extraction and RT-qPCR Analysis

*Candida albicans* cells were harvested using centrifugation at 4000 rpm and 4°C, and cell pellets were resuspended in 200 μL ice-cold extraction buffer [0.1 M LiCl, 0.1 M Tris-HCl (pH 7.5), 5% SDS, 2% β-mercaptoethanol]. The mixtures were transferred to a new microcentrifuge tube that contained 0.3 g of acid-washed glass beads and PCIA (Phenol-Chloroform-isoamyl alcohol) pH 4.5, vortexed at full speed for 5 min and placed immediately on ice for precipitation. Nucleic acid pellets were collected by centrifugation, washed with 70% ethanol, air-dried, and then dissolved in 100 μL nuclease-free water. DNA contaminants were removed with RNase-free DNase I (Promega). The 20-μL reaction mixture included 2^∗^SYBR Green Master Mix (High ROX premixed), 5 μL of first-strand cDNA reaction mixture, and 0.4 μM primers. The amplification program and analysis were performed according to the manufacturer’s instructions. Quantitative polymerase chain reaction (qPCR) was performed using the SYBR Green Master Mix (High ROX Premixed) (Vazyme, Nanjing, China) using the primers CBO284 and CBO285 for TEC1, CBO282 and CBO283 for EFG1, CBO280 and CBO281 for BRG1, CBO278 and CBO279 for BCR1, CBO286 and CBO287 for NDT80, CBO288 and CBO289 for ROB1, CBO292, and CBO293 for HWP1 (**Table 2**).

**Table 2:** Oligonucleotides used in this study.

Primers		Oligonucleotide sequence
*BCR1*	CBO278	TACACCTAAAAGAAGACCCGAACC
	CBO279	CCGTGTTCATATTGGTGTTGGT
*BRG1*	CBO280	CACCATCTGTAACACATCAAGGTC
	CBO281	CACCTGTGACATCTGTTAGTTGAC
*EFG1*	CBO282	AGTACCTATCCCACCACATGTATC
	CBO283	GTTGTTACTCGTGGTCTGATTCC
*TEC1*	CBO284	GTCCTATTTTCAACAGTCACGAGG
	CBO285	CTTATTCTCTTTGTGGCTGGGAG
*DDT80*	CBO286	AATCTACCCTGCAGTTCCTTCAG
	CBO287	GTACTCTTGGTAGTAGTTTCCCCT
*ROB1*	CBO288	TATATCACCTCCACCACTGTTACC
	CBO289	GCCATATTTGATTTCCCACCAGG
*HWP1*	CBO292	TCTCTACGACTGAAGGTGCTATTC
	CBO293	CCAATAATAGCAGCACCGAAAGTC


### Ethics Statement

All animal experiments were approved by Institut Pasteur of Shanghai Chinese Academy of Sciences (IPS) IACUC [A20150013]. All animal experiments were carried out in strict accordance with the regulations in the Guide for the Care and Use of Laboratory Animals issued by the Ministry of Science and Technology of the People’s Republic of China. Animals were euthanized by carbon dioxide inhalation.

### Mouse Vaginal Infection

Nine to ten-week-old female C57BL/6 mice were purchased from Vital River Laboratory Animal Technology Co., Ltd. The mouse model of vaginal infection was established as described elsewhere (Enjalbert et al., 2009; Pietrella et al., 2011). Three days prior to infection a pseudoestrus condition was induced in the mice by administration of subcutaneous injection of 0.2 mg estradiol valerate in 100 μL of sesame oil (Sigma–Aldrich). The injection was administered weekly throughout the duration of the experiment. Mice were anesthetized with 0.5 mL chloral hydrate per 100 g body weight and inoculated with 20 μL of 5 × 10^8^ CFU/mL *C. albicans* administered by mechanical pipette into the vaginal lumen, close to the cervix.

### Vaginal Cell Collection

Vaginal lavage was performed on the mice using 500 μL PBS and the fungal burden in lavage fluid was assessed. Vaginal lavage obtained at different time-points after infection were treated with protease inhibitors and centrifuged at 600 × *g*. Supernatants were recovered and stored at -20°C, and the pellets were fixed with 4% paraformaldehyde (PFA). To assess the number of polymorphonuclear cells in vaginal wash fluid, the cells were incubated with rat anti-mouse Ly6G-Percp Cy5.5, CD4-FITC, CD8-APC, F4/80-PE for 30 min on ice. Then the cells were analyzed by flow cytometry.

### Measurement of Cytokines in Mice Cervical-Vaginal Lavage Solution

Supernatants from vaginal lavage obtained from co-infected and mono-infected mice were tested for expression levels of IL-1β, IL-2, IL-6, TNF-α, IFN-γ, and IL-17. Cervical-vaginal lavage solution were collected and homogenized in 500 μl of cold PBS supplemented with protease inhibitors (1 mM phenylmethylsulfonyl fluoride, 1 μM leupeptin, and 0.3 μM aprotinin) using a Dounce homogenizer. The samples were then centrifuged at 4000 rpm, and the resulting supernatant was stored at -80°C. IL-1β, IL-2, IL-6, TNF-α, and IFN-γ levels in the solution were measured with Ready-SET-GO ELISA kits (eBioscience). The level of IL-17 protein was measured using an enzyme-linked immunosorbent assay (ELISA) kit (eBioscience, San Diego, CA, United States), according to the manufacturer’s directions. All samples were measured in triplicate according to the manufacturer’s protocol. The analysis of surface molecules, intracellular IL-17 in vaginal, and lymph node cells was performed by flow cytometry.

### Histological Analysis

For histological analysis, the mice were sacrificed, and the vaginal tissue were removed and immediately fixed in 4% (v/v) neutral buffered formalin for 24 h. The vaginal specimens were then dehydrated, paraffin-embedded, and 3–4 μm thick sections prepared. The sections were stained with periodic acid-Schiff stain (PAS).

### Statistical Analysis

Results were compared using the non-parametric Kruskal–Wallis test, non-parametric Mann–Whitney Test, or the paired *t*-test using Prism software (GraphPad). *P-*values < 0.05 were considered to be statistically significant.

## Results

### Hyphal Development Was Found to Be Attenuated in Clinical Isolates of *C. albicans* from Patients with RVVC Having Co-infection with *S. agalactiae*

**FIGURE 1:**
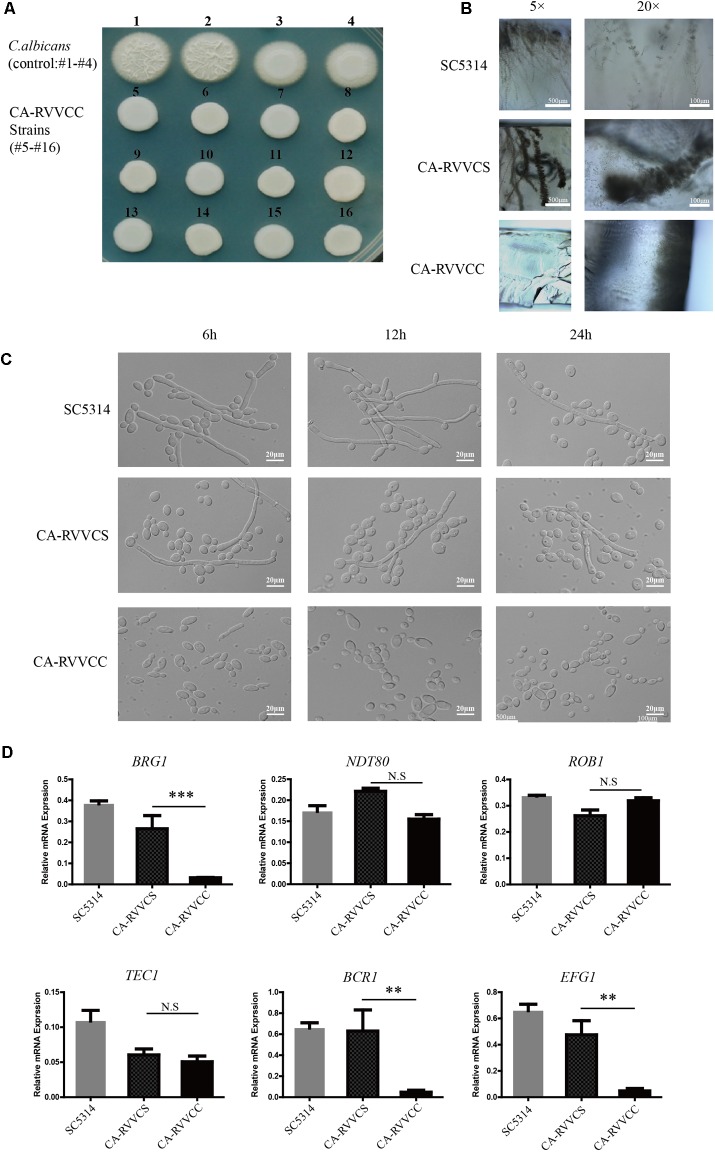
*Candida albicans* strains isolated from patients with RVVC co-infected with *Streptococcus agalactiae* show defective hyphae formation. *C. albicans* strains isolated from patients with RVVC with or without *S. agalactiae* co-infection, were cultured at 37°C. **(A)** *C. albicans* cells morphology on Spider plate at 37°C after 3 days (NO.1-2:SC5314 strains; NO.3-4:CA-RVVCS strains; NO.5-16: CA-RVVCC strains). **(B)** Hyphal morphology was observed in longitudinal sections of agar under the microscope. **(C)** SC5314, CA-RVVCS and CA-RVVCC cells were cultured in YPD medium at 37°C for 6 h, 12 h and 24 h to observe cells morphology the microscope. **(D)** The mRNA levels of transcriptional regulators *EFG1, NDT80, ROB1, BRG1, TEC1*, and *BCR1* were measured by RT-qPCR. (^∗^*P* < 0.05, ^∗∗^*P* < 0.01, ^∗∗∗^*P* < 0.001).

*Candida albicans* can lead to mucosal infections in VVC and RVVC. Interestingly, we found that *C. albicans* strains isolated from patients with RVVC hyphal development were different on Spider plate (**Supplementary Figures S1A,B**). This difference may be the results of differences in the duration of infection and treatment. We named *C. albicans* strains isolated from patients with RVVC infection without bacterial infection as CA-RVVCS and named *C. albicans* strains isolated from patients of RVVC with *S. agalactiae* co-infection as CA-RVVCC. We further analyzed clinical strains hyphal development on Spider medium. Interestingly, we found that 82% (28/34) CA-RVVCC strains hyphal development were deficient, but 47% (16/34) CA-RVVCS strains hyphal development were deficient (**Supplementary Figure S2A**). To explore CA-RVVCC strains hyphal development, *C. albicans* cells isolated from clinical samples were cultivated on Spider plate at 37°C for 3 days to induce hyphal development. As a control, the SC5314 strains (*n* = 2) and CA-RVVCS strains (*n* = 2) were cultivated on Spider plate. We found that compared with the control strains, the colonies of CA-RVVCC strains (*n* = 12) exhibited fewer wrinkles suggesting that hyphal development of CA-RVVCC strains appears to be a deficient (**Figure 1A**). On microscopic examination, the colonies of CA-RVVCC strains also exhibited a smooth appearance, which indicated impairment of hyphal development. *C. albicans* on the Spider plate at 37°C induced hyphal development with upward growth in the air and downward growth invading the agar. This invasive growth of *C. albicans* hyphae can induce host tissues damage triggering immune defense responses. We observed the hyphal growth of CA-RVVCC strains in the agar to further assess these strains hyphal development. Next, the agar was cut longitudinally to observe the invasive growth of *C. albicans* and hyphal morphology by microscopy. On microscopic examination, the CA-RVVCC strains hyphal morphology showed shorter and fewer in the agar, when compared with that of SC5314 and CA-RVVCS strains, suggesting that CA-RVVCC strains hyphal invasive growth were impaired (**Figure 1B**). To examine whether the phenotype has requirements for growth medium, we further assessed hyphal development in YPD media. These isolates were cultured in YPD medium at 37°C for 6 h, 12 h and 24 h to observe hyphal development. At different time-points hyphal morphology was observed by the microscopy. The majority of cells of the SC5314 and CA-RVVCS strains were found to have hyphal morphology. In contrast, the majority of cells of the CA-RVVCC strains were found to have yeast-like morphology, and hyphal development was found to be severely attenuated at 6 h, 12 h and 24 h (**Figure 1C**). The transcription factors *EFG1, NDT80, ROB1, BRG1, TEC1*, and *BCR1* have been shown to be involved in hyphal development of *C. albicans* (Nobile et al., 2012). In order to identify factors contributing to the hyphal deficient phenotypes, the transcriptional levels of *EFG1, NDT80, ROB1, BRG1, TEC1*, and *BCR1* were assessed on RT-qPCR to identify genes that regulate hyphal development. The hyphal development transcription factors *EFG1, BRG1* and *BCR1* were found to be downregulated in CA-RVVCC strains when compared with SC5314 and CA-RVVCS strains (**Figure 1D**). These findings suggested that *S. agalactiae* co-infection may have attenuated the hyphal development of *C. albicans* in VVC.

### *S. agalactiae* Inhibits Hyphal Development of *C. albicans in Vitro*

**FIGURE 2:**
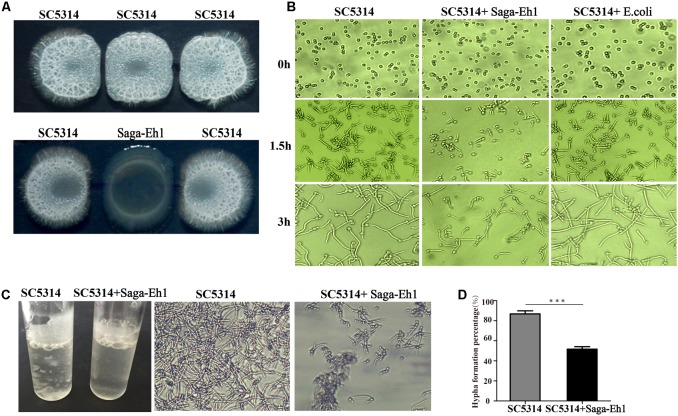
*Streptococcus agalactiae* inhibits hyphal development in *C. albicans* under different culture conditions. An overnight culture of *C. albicans* strain SC5314 was diluted 1:50 in the indicated medium at 37°C and the cells collected for examination of cells morphology **(A)**, SC5314 (2 × 10^5^CFU/μL) in culture medium (5 μL) and Saga-Eh1 (1 × 10^7^ CFU/μL) in culture medium (5 μL) were dropped on Spider plate and cultured at 37°C for 3 days to observe the effect of Saga-Eh1 on hyphal development in SC5314. **(B)** SC5314 was cultured with/without Saga-Eh1 culture supernatant at 37°C and collected at 0 h, 1.5 h and 3 h to examine hyphal morphology under the microscope. **(C)** SC5314 was cultured at 37°C for 6 h in Spider medium with/without Saga-Eh1 culture supernatant to examine hyphal aggregation. **(D)** SC5314 was cultured at 37°C for 6 h in Spider medium with/without Saga-Eh1 culture supernatant to examine the percentage of hyphal formation under the microscope. All experiments were performed in triplicate. (^∗^*P* < 0.05, ^∗∗^*P* < 0.01, ^∗∗∗^*P* < 0.001).

Hyphal development is an important factor in the pathogenesis of *C. albicans*. We found that the Saga-Eh1 strain could inhibit hyphal development in some CA-RVVCS strains (**Supplementary Figure S2B**). To investigate further the interaction of *S. agalactiae* and *C. albicans*, we studied hyphal development in *C. albicans* after co-cultured with Saga-Eh1. The SC5314 cells were harvested from overnight cultures and re-inoculated in YPD medium. Hyphal development in *C. albicans* was examined in SC5314 cultivated on Spider plate at 37°C for 3 days, with or without Saga-Eh1. When the Saga-Eh1 and SC5314 were grown adjacent to one another, we found that compared with the growth of SC5314 alone, there was a striking growth suppression of *C. albicans* in the presence of *S. agalactiae*. SC5314 co-culturing with Saga-Eh1 colonies exhibited fewer wrinkles when compared with SC5314 hyphal development on Spider plate suggesting that under a condition, *S. agalactiae* is able to depress growth of SC5314 (**Figure 2A**). This result indicated that Saga-Eh1 inhibited SC5314 hyphal development via non-contact suppression. Next, we analyzed the process of morphological development from yeast to hyphae in SD+10% FBS. Saga-Eh1 overnight culture supernatant was 10 times concentrated and mixed with SD+10% FBS (1:10) to culture SC5314. At the same time, SD+10% FBS mixed with 10 times concentrated *E. coli* culture supernatant (1:10) was the control group to observe hyphal morphological development in SC5314. Hyphal elongation in SC5314 was found to be attenuated at 3 h after mixing with Saga-Eh1 culture supernatant when compared with normal SC5314 hyphal development in *E. coli* culture supernatant (**Figure 2B**). We further examined hyphal development in different media. *C. albicans* strain SC5314 was co-cultured with *S. agalactiae* strain Saga-Eh1 in Spider, YPD+10% FBS, DMEM+10% FBS, and SD+10% FBS medium at 37°C for 4 h to induce hyphal development, followed by microscopic examination of hyphal morphology. The results showed that length of *C. albicans* hyphae were short in different culture media (**Supplementary Figure S3A**). These findings indicated that *C. albicans* strain SC5314 hyphal development was attenuated upon co-culture with *S. agalactiae*.

Hyphal aggregation is another important characteristic of *C. albicans*. To evaluated *C. albicans* hyphal aggregation, *C. albicans* strain SC5314 was cultured in SD+10% FBS with and without 10 times concentrated Saga-Eh1 culture supernatant in tube at 37°C for 6 h to observe SC5314 hyphal aggregation. Surprisingly, visible massive precipitation was observed in the absence of Saga-Eh1 culture supernatant tube, while only small particular precipitates were observed in the tube with Saga-Eh1 culture supernatant. Differences in the morphology of *C. albicans* cultured with and without Saga-Eh1 culture supernatant were also observed during microscopic examination (**Figure 2C**). Furthermore, we investigated the percentage of *C. albicans* hyphal formation in the *in vitro* co-culture. *C. albicans* was incubated for 6 h in Spider media, with gentle shaking of samples at 37°C. Hyphal and yeast counts were performed in 200 *C. albicans* cells under a microscope. We found that hyphal formation with co-culture of 10 times concentrated Saga-Eh1 culture supernatant was lower than that in the absence of Saga-Eh1 culture supernatant (50.29 ± 8.58% vs. 86.80 ± 9.34%, *P* < 0.001), which indicated that *S. agalactiae* suppressed *C. albicans* hyphal formation *in vitro* (**Figure 2D**). Collectively, these results confirmed that *S. agalactiae* could suppress *C. albicans* hyphal development *in vitro*.

### *S. agalactiae* Inhibits *EFG1*-Induced Hwp1 Production to Attenuate *in Vitro* Hyphal Development in *C. albicans*

**FIGURE 3:**
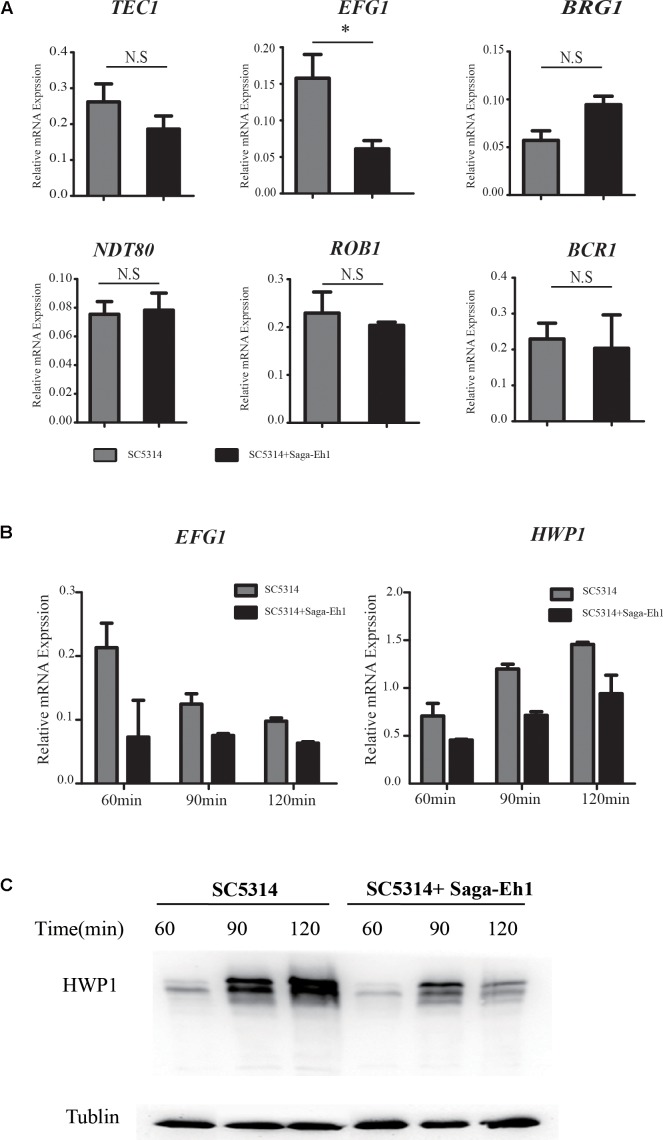
*Streptococcus agalactiae* inhibits *EFG1*-induced Hwp1 production during hyphal development on co-culture with *C. albicans.* An overnight culture of SC5314 was diluted 1:50 with SD+10% FBS at 37°C, and cells were collected to determine **(A)** the transcriptional regulators *EFG1, NDT80, ROB1, BRG1, TEC1*, and *BCR1* mRNA levels were measured by RT-qPCR. **(B)** An overnight culture of SC5314 was diluted 1:50 in SD+10% FBS and cultured at 37°C; cells were collected at 30 min, 60 min and 90 min. The mRNA levels of *EFG1* and *HWP1* were measured by RT-qPCR. **(C)** The protein expression of HWP1 was measured by Western blotting (^∗^*P* < 0.05, ^∗∗^*P* < 0.01, ^∗∗∗^*P* < 0.001).

The transcriptional network is critical to hyphal development in *C. albicans*. To explore the underlying mechanism of impaired hyphal development, we assessed the mRNA expressions of transcription factors associated with hyphal development using reverse transcription quantitative polymerase chain reaction (RT-qPCR). We found that the transcription level of *EFG1* was decreased significantly in *C. albicans* co-cultured with *S. agalactiae*, when compared with that in *C. albicans* cultured without *S. agalactiae*, while the mRNA levels of other transcription factors were comparable to those of the controls (**Figure 3A**). Hyphal wall protein (Hwp1) extends to fungal growth in the host, as shown by the presence and absence of Hwp1 on hyphae and yeast (Fan et al., 2013). Hwp1 is a transglutaminase substrate which functions as an adhesin and plays an important role in the pathogenesis of candidiasis (Sundstrom et al., 2002; Martin et al., 2011; Fan et al., 2013; Staab et al., 2013). To investigate further the underlying mechanism of the effect of *S. agalactiae* on hyphal development in *C. albicans*, the expressions of transcription factors *EFG1* and hyphal development marker *HWP1* were assessed on RT-qPCR. Serial RT-qPCR at various time-points showed that the mRNA expressions of *EFG1* and *HWP1*, after hyphal induction were significantly decreased on co-culture with *S. agalactiae* (**Figure 3B**). Moreover, the expression of Hwp1 in the co-culture group determined on Western blotting analysis at 60 min, 90 min and 120 min was lower than that in the control group (**Figure 3C**). Together, these findings suggest that co-culture with *S. agalactiae* attenuates hyphal development of *C. albicans* by downregulating *EFG1-*Hwp1.

### *S. agalactiae* Promotes *C. albicans* Colonization of Mucosa in Mouse Model of VVC

**FIGURE 4:**
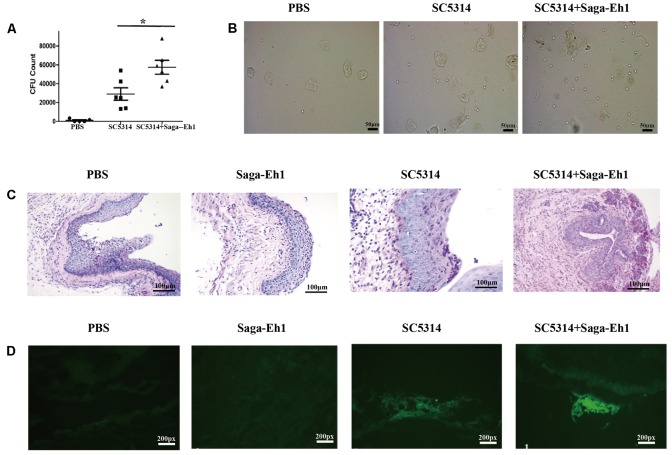
Co-infection of vaginal mucosa with *C. albicans* and *S. agalactiae* facilitates *C. albicans* colonization. Mice (*n* = 6 per group) were infected with SC5314 with or without Saga-Eh1 for 5 days. Mice were then sacrificed, and cervical-vaginal lavage solution was douched by 500 μL PBS. **(A)** The pellets of mice cervical-vaginal lavage solution were resuspended in 100 μL PBS and plated on YPD (1% ampicillin and 1% gentamicin). The fungal burden was determined by CFU counting. **(B)** Neutrophil cells counts were determined under the microscope. **(C)** SC5314 colonized on mice vaginal mucosa were detected by PAS staining. **(D)** SC5314-GFP colonizing murine vaginal mucosa was detected on frozen sections by fluorescence microscope; recorded data was based on at least three different points in three different sections of each vagina. (^∗^*P* < 0.05, ^∗∗^*P* < 0.01, ^∗∗∗^*P* < 0.001).

Hyphal formation and adherence are pivotal for biofilm formation by *C. albicans*, which is involved in the pathogenesis of many infections (Andes et al., 2004; Fox and Nobile, 2012). Different components of the immune system identify hyphae by different mechanisms, which are pivotal for inducing host defense response (Gow, 2013). To explored the role of *S. agalactiae* in the pathogenesis of *C. albicans* infection, a mouse model of VVC was established. Vaginal lumen of mice were inoculated with SC5314 and Saga-Eh1 (MOI = 10). Five days post-inoculation, 500 μL PBS was used to perform mice vaginal lavage. This lavage fluid was plated on YPD and cultured to assess mice vaginal fungal burden by CFU counting. The vaginal fungal burden in mice infected with *C. albicans* and Saga-Eh1 was greater than that in mice without Saga-Eh1 (5.75 × 10^4^ ± 420 per mouse vs. 2.9 × 10^4^ ± 191 per mouse, *P* < 0.05) (**Figure 4A**). Neutrophils are an important immune cell and were detected to evaluated host immune defense reaction. In VVC mouse vaginal lumen, neutrophil cells counts in vaginal lavage fluid showed significant difference between-group differences (1234 ± 122.5 per mouse vs. 769 ± 130.3 per mouse, *P* < 0.05) (**Figure 4B**). Furthermore, histopathological examination of vaginal mucosa stained with PAS (Periodic acid–Schiff staining, PAS) on day 5 post-inoculation showed massive *C. albicans* colonization in VVC mice co-infected with Saga-Eh1, when compared with mice without Saga-Eh1 co-infection (**Figure 4C**). To confirm *C. albicans* colonization on vaginal mucosa, SC5314-GFP strain was used to infect mice to establish a mouse model of VVC. On fluorescence microscopy, more number of colonies of SC5314-GFP were observed on vaginal mucosa of mice co-infected with Saga-Eh1 as compared to that in vaginal mucosa of the control mice (**Figure 4D**). Analysis of the total photon emission showed a significant reduction in the fungal load on vaginal mucosa of mice co-infected with Saga-Eh1 as compared to that in vaginal mucosa of control mice on day 5 post-inoculation. Similar results were observed on PAS staining in the same mice.

Colonization of the vaginal mucosal by *C. albicans* is known to depend on adhesion of *C. albicans* and on the host-immune clearance (Gow et al., 2012). The adherence of fungal cells to host epithelial cells during the initial contact is important because it determines the subsequent fungal-host interactions.

The adhesion of the fungus to epithelial cells is a complex process mediated by hyphal-associated factors, such as Hwp1 and Als3 (Martin et al., 2011; Mech et al., 2014; Moyes et al., 2015). Human epithelial colorectal adenocarcinoma cells (Coca2 cells) and human epidermoid carcinoma cells (A431 cells) were cultured in Dulbecco’s Modified Eagle medium (DMEM)+10% FBS medium at 37°C; then *C. albicans* strain SC5314 was cultured with or without Saga-Eh1 in transwell at 37°C. After 2 h of co-culture, no significant difference was observed in the counts of adherent cells between the two groups. This indicated that *S. agalactiae* did not affect adhesive ability of *C. albicans* (**Supplementary Figure S3B**).

### Vaginal Mucosa TH17 Immune Response Is Decreased by *S. agalactiae* Co-infection

**FIGURE 5:**
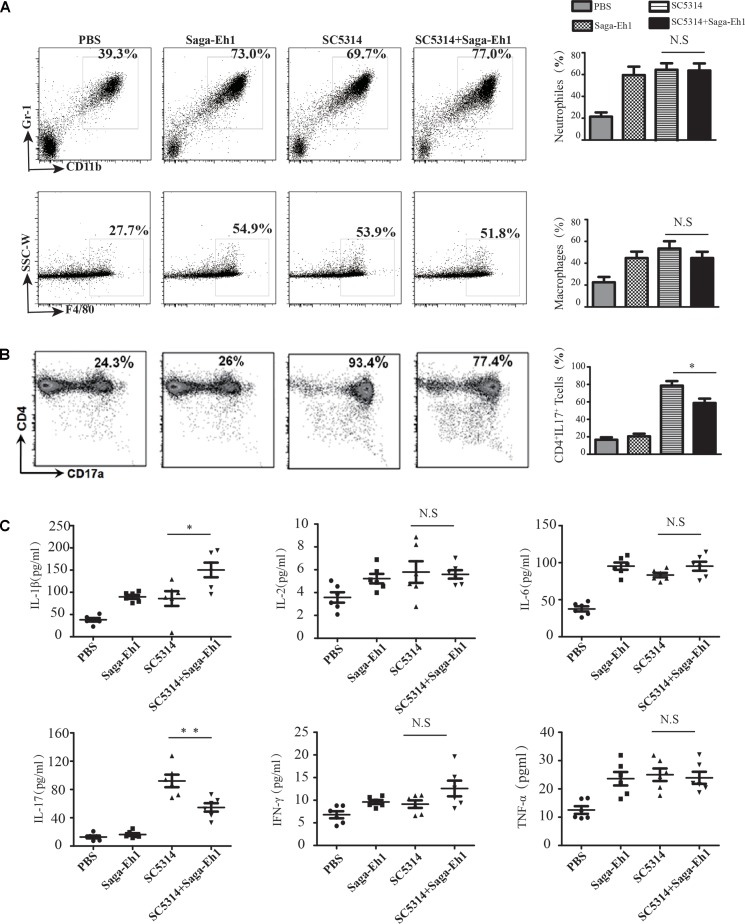
Host vaginal mucosal TH17 immune response was attenuated by *C. albicans* and *S. agalactiae* co-infection. Mice (*n* = 6 per group) were infected with SC5314 with or without Saga-Eh1 for 5 days. Mice cervical-vaginal lavage fluid was collected and mice were sacrificed to collect lymphocytes, which were stained with mAbs. **(A)** Macrophages and neutrophil cells populations in cervical vaginal lavage solution were determined by flow cytometry. **(B)** TH17 T cells population in cervical vaginal lavage solution was determined by flow cytometry. **(C)** The levels of IL-1β, IL-2, IL-6, TNF-α, IFN-γ, and IL-17 were determined by ELISA (^∗^*P* < 0.05, ^∗∗^*P* < 0.01, ^∗∗∗^*P* < 0.001).

Neutrophils and TH17 cells play an important role in the immune response against colonization of vaginal mucosal surface by *C. albicans* (Pietrella et al., 2011; Hopke et al., 2016; Salvatori et al., 2016). To explore host immune responses induced by *C. albicans* and *S. agalactiae* co-infection, Saga-Eh1and SC5314 were inoculated into the vaginal mucosa of mice and the immune responses were monitored using flow cytometry. The results showed presence of different types of cells in the vaginal fluid of the infected mice, especially neutrophils and epithelial cells. On day 5 post-inoculation, vaginal mucosal immune cells were detected by flow cytometry. The results showed that the percentage of neutrophils cells increased from 65.53 ± 14.45% to 71.67 ± 15.92% (*P* > 0.05), and the percentage of macrophages decreased from 39.53 ± 16.37% to 35.80 ± 13.93% (*P* > 0.05); however, these percentages were not significantly different between the co-infected mice and controls (**Figure 5A**). During the early phase of vaginal mucosal infection, neutrophils cells were recruited and extravasated using pro-inflammatory mediators, which was followed by activation of TH17 response (Kolaczkowska and Kubes, 2013). TH17 cells belong to a lineage that is different from the Th1 and Th2 cells, and these are characterized by the production of IL-17. We observed that the number of TH17 T lymphocytes were significantly decreased in mice co-infected with Saga-Eh1 as compared to that in control mice (89.28 ± 3.38% vs. 69.93 ± 5.71%, *P* < 0.05) (**Figure 5B**). However, no significant differences were observed with respect to CD4+ T cells (20.83 ± 7.66% vs. 22.6 ± 6.96%, *P* > 0.05), and CD8+ T cells (5.62 ± 4.43% vs. 3.395 ± 1.94%, *P* > 0.05) (data not shown).

The IL-17 secreted by activated TH17 T cells has been shown to play a critical role in protecting against mucosal infections, and particularly against mucosal candidiasis (Pietrella et al., 2011; Puel et al., 2011). The IL-17 response correlated with the hyphal formation ability of *C. albicans*, which stimulates macrophages, neutrophils, and other innate immune cells, hence priming the host-innate immune response and inducing cytokine production for priming the TH17 response. We further investigated the related inflammatory cytokines in a mouse model of vaginal infection. Production of cytokines was higher in the cervical-vaginal lavage solution of mice co-infected with Saga-Eh1 compared with those given mono-infection with SC5314 as IL-1β (114.65 ± 2.66 pg/mL vs. 122.6 ± 6.96 pg/mL, *P* < 0.05); IL-6 (84.60 ± 1.05 pg/mL vs. 86.01 ± 2.21 pg/mL, *P* > 0.05); and TNF-α (78.83 ± 3.43 pg/mL vs. 81.6 ± 4.96 pg/mL, *P* > 0.05)] (**Figure 5C**). Activated TH17 cells produced more IL-17 to enable *C. albicans* clearance. However, post-challenge there was decreased production of IL-17 (42.10 ± 1.43 pg/mL vs. 31.6 ± 4.96 pg/mL, *P* < 0.05) in the vaginal washes from co-infected Saga-Eh1 mice as compared to that in mice with mono-infection SC5314 (**Figure 5C**). Collectively, these results demonstrated that TH17 T cells immune response in vaginal mucosa was decreased due to *S. agalactiae* co-infection.

## Discussion

*Candida albicans* is the dominant pathogen in VVC and RVVC. Our results indicate that *S. agalactiae* inhibited *C. albicans* hyphal development by decreasing expression of EFG1/Hwp1. In the mouse model of VVC, co-infection with *S. agalactiae* promoted more *C. albicans* colonization of the vaginal mucosa. Vaginal mucosal colonization by *C. albicans* is known to depend on adhesion of *C. albicans* and on the host immune clearance (Gow et al., 2012). Although *S.agalactiae* inhibited hyphal development in *C. albicans* and decreased *C. albicans* mucosal colonization, this co-infection severely attenuates vaginal mucosa TH17 T cells immune response and decreased host immune clearance facilitating colonization by *C. albicans*.

Hwp1 is probably required by *C. albicans* to establish a firm footing on the mucosal surface, which enables other hyphal factors to invade and cause damage. In the current study, decrease in Hwp1 protein expression was accompanied by increase in mucosal colonization by *C. albicans*. Interestingly, co-infection with *S. agalactiae* increased colonization of mice vaginal mucosa by *C. albicans*. This intriguing phenotype indicates that the increased *C. albicans* colonization was due to impaired host clearance.

Previous studies have shown interaction of *C. albicans* with different bacteria; however, the mechanism by which this interaction affects the host immune response is not clear. Although an impaired innate immune response contributes to chronicity of infection, a characteristic feature of chronic *C. albicans* infection is the lack of a robust TH17 T cell response (Rouse and Sehrawat, 2010; Pietrella et al., 2011). It is well-established that vaginal mucosal immune response is specific in nature and that TH17 immune response is involved in the clearance of *C. albicans*. Hyphal formation and adherence are pivotal for biofilm formation by *C. albicans*, which is involved in the pathogenesis of many infections. Hyphal development is important in triggering host adapt immune response. In mucosal candidiasis, activated TH17 T cells secreting IL-17 play a critical role in protecting against mucosal infections. This process is mediated primarily by inflammatory cytokines, including IL-1β, IL-6, TNF-α, and IL-17. IL-17 plays a central role in both systemic and local (oral) immunity against infection with *C. albicans* (Huang et al., 2004; Conti et al., 2009). In this study, levels of IL-17 in the cervical-vaginal lavage fluid from VVC mice were significantly decreased following co-infection with *S. agalactiae* and *C. albicans*. These results suggest that *S. agalactiae* and *C. albicans* co-infection may be an effective intervention in the host evolution of pathogenicity and host-parasite relationships, by downregulating the levels of TH17 T cells immune response. Analyses of TH17 T cells isolated from cervical-vaginal lavage solution from mice with VVC demonstrated decreases in the TH17 T cell population and weakened IL-17 production. We speculated that inhibition of hyphal development in *C. albicans* resulted in decreased damage to vaginal mucosa, and lack of host stimulation to release more and strong cytokines to induce more CD4+ T cells to differentiate into TH17 T cells. The defective TH17 T cells immune response could not effectively clear *C. albicans;* however, which promoted colonization of vaginal mucosa by *C. albicans*.

We isolated *S. agalactiae* mixed with *C. albicans* in about 5% of patients with RVVC, and found that *S. agalactiae* could inhibit *C. albicans* hyphal development, which is required for *C. albicans* virulence and invasiveness. RVVC is a chronic mucosal infection; its pathogenesis and treatment are not clear and need further study (Fidel et al., 2004). Recent efforts against RVVC infections have focused on measures to ameliorate and balance the vaginal micro-ecology. We speculate that *S. agalactiae* and *C. albicans* co-infection attenuates host TH17 T-cell mediated immune response against *C. albicans*, which facilitates long-term colonization of the vaginal mucosa but delays mucosal infection. This may contribute to latent infection with *C. albicans* in patients with RVVC and may be a potential pathogenic mechanism of RVVC. It is worth mentioning that our results showed that *S. agalactiae* inhibited hyphal development in *C. albicans* even in the absence of mutual contact. This finding indicates that S. *agalactiae* may secrete some active compounds to inhibit hyphal development in *C. albicans* and implies the possibility of finding a new method to control hyphal development in *C. albicans*.

## Author Contributions

X-YY, FF, W-NK, Q-KX, D-HW, X-QC, Y-MH, L-HH, JG, A-PZ, Y-HX, and L-JN conducted the experiments. Y-FY and W-JW planned and supervised the experiments. X-YY and FF wrote the paper. All authors gave intellectual input to the study and approved the final version of the manuscript.

## Conflict of Interest Statement

The authors declare that the research was conducted in the absence of any commercial or financial relationships that could be construed as a potential conflict of interest.

## Supplementary Material

**FIGURE S1 |** Hyphal development in CA-RVVC strains on Spider plate. An overnight culture of CA-RVVC strains was diluted 1:50 with the indicated medium at 37°C. Cells cultured in YPD medium were collected for examination of morphology. **(A)** CA-RVVC (*n* = 16) (2 × 10^5^ CFU/μL) in culture medium (5 μL) were dropped on Spider plate cultured at 37°C for 3 days and hyphal development observed; **(B)** CA-RVVC (*n* = 6) (2 × 10^5^ CFU/μL) in culture medium (5 μL) were dropped on Spider plate cultured at 37°C for 5 days and hyphal development was observed.

**FIGURE S2 |** Hyphal development in CA-RVVCS strains on Spider medium. An overnight culture of CA-RVVCS was diluted 1:50 with the indicated medium at 37°C. Cells cultured in YPD medium were collected for examination of morphology. **(A)** CA-RVVCS (*n* = 16) (2 × 10^5^ CFU/μL) in culture medium (5 μL) were dropped on Spider plate cultured at 37°C for 3 days and hyphal development were observed. **(B)** CA-RVVCS (2 × 10^5^ CFU/μL) in culture medium (5 μL) and Saga-Eh1 (1 × 10^7^ CFU/μL) in culture medium (5 μL) were dropped on Spider plate cultured at 37°C for 5 days to observe the effect of Saga-Eh1 on hyphal development in CA-RVVCS.

**FIGURE S3 |***Streptococcus agalactiae* inhibits *C. albicans* hyphal development in different culture media. **(A)** An overnight culture of SC5314 was diluted 1:50 with the indicated medium at 37°C. Cells cultured in different medium were collected for examination of morphology. *C. albicans* strain SC5314 was co-cultured with *S. agalactiae* strain Saga-Eh1 in transwell in Spider, YPD+10% FBS, DMEM+10% FBS, SD+10% FBS medium at 37°C for 4 h. Then cells were collected and cell morphology examined by microscopy. **(B)** AV431/Coca2 cells (5 × 10^6^) and *C. albicans* cells (300 CFU) were co-cultured in lower chamber in complete DMEM media; *S. agalactiae* cells (3000CFU) were added to the upper part of the transwell chamber, at 37°C for 2 h. Subsequently, AV431/Coca2 cells were collected to observe *C. albicans* cells adhesion by colony counts.
