# Supplementary figures and images for: Streptococcus agalactiae Inhibits Candida albicans Hyphal Development and Diminishes Host Vaginal Mucosal TH17 Response
Source: Front Microbiol. 2018 Feb 23;9:198. doi: 10.3389/fmicb.2018.00198 (PMC5829043; doi:10.3389/fmicb.2018.00198)

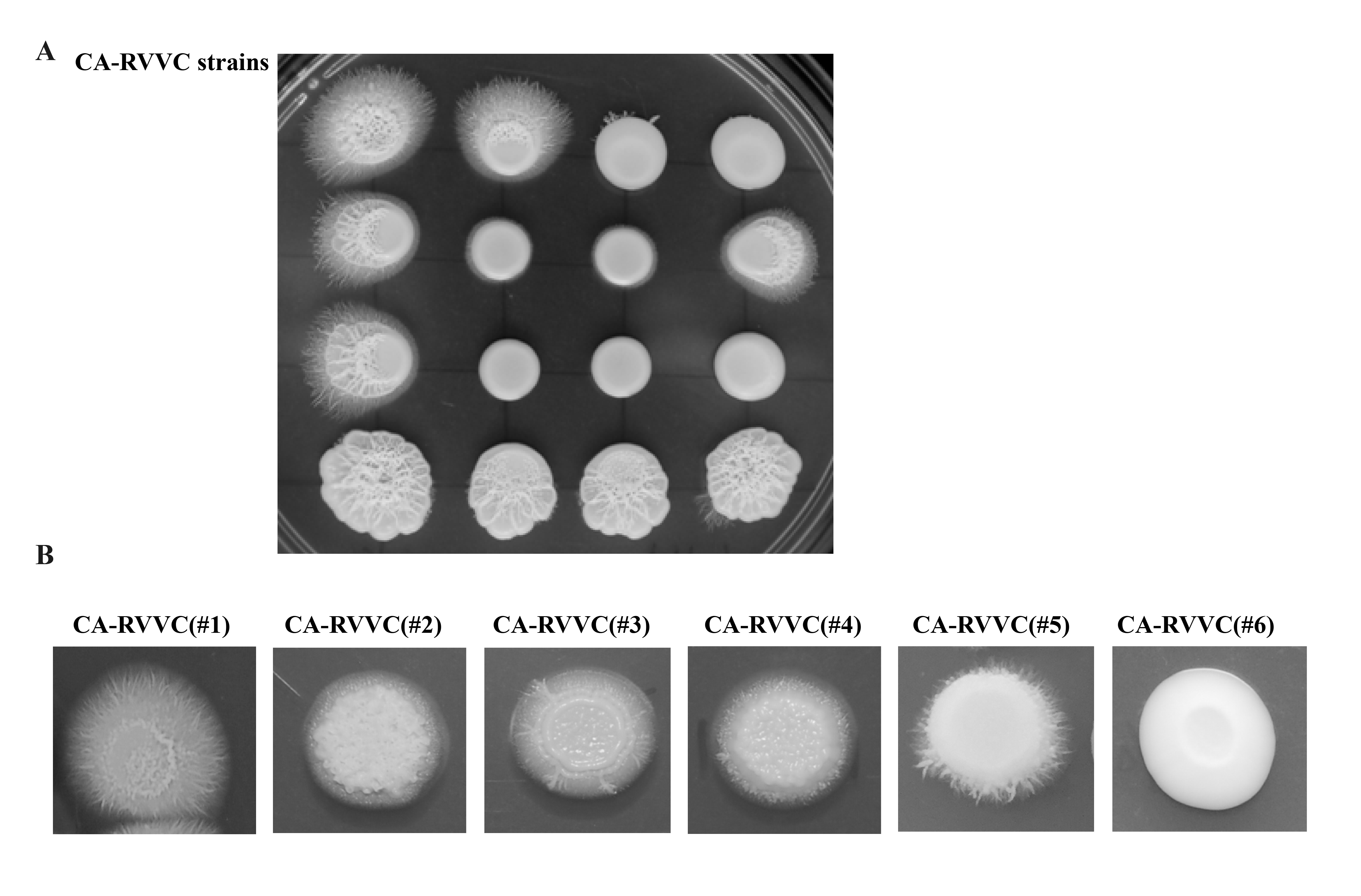

Supplement: Supplementary file 1 [file Image_1.tif]

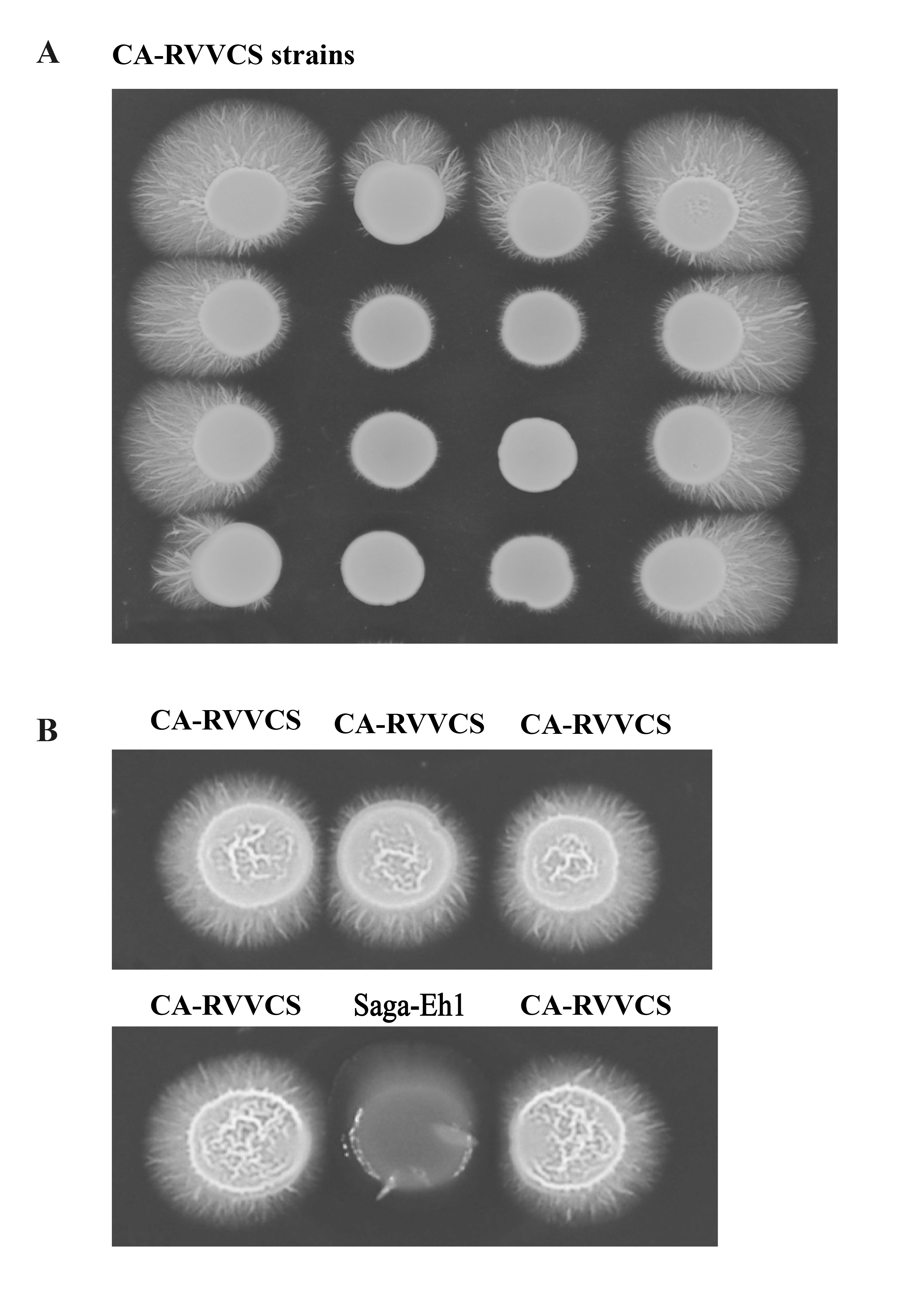

Supplement: Supplementary file 2 [file Image_2.tif]

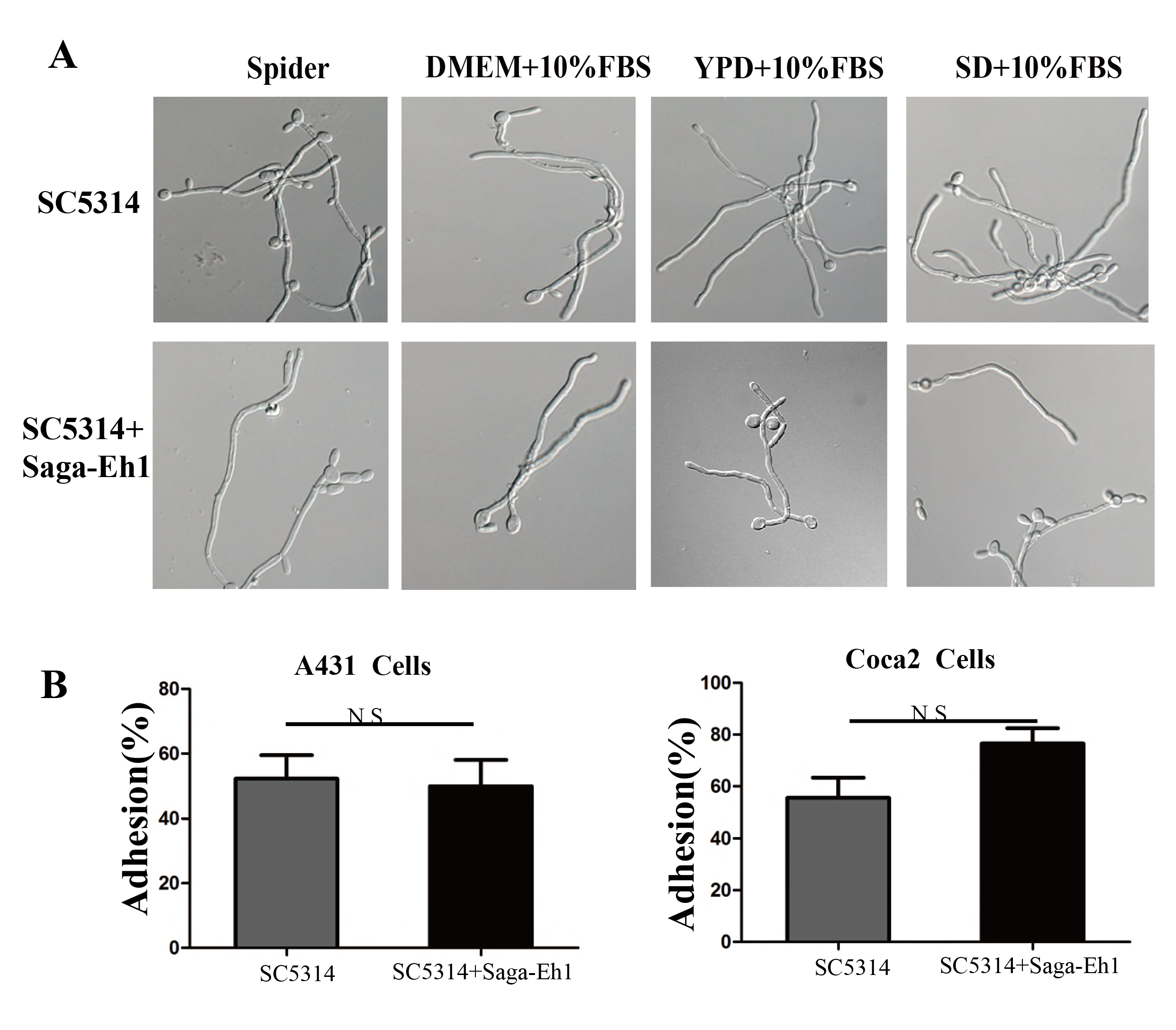

Supplement: Supplementary file 3 [file Image_3.tif]
